# Benefit-risk assessment of traditional Chinese medicine preparations of sinomenine using multicriteria decision analysis (MCDA) for patients with rheumatoid arthritis

**DOI:** 10.1186/s12906-023-03864-6

**Published:** 2023-02-06

**Authors:** Gao Xiang, Min Gao, Huirong Qin, Xiaolan Shen, Huilian Huang, Xiaoqiang Hou, Zhitao Feng

**Affiliations:** 1 The First College of Clinical Medical Sciences, China Three Gorges University, Yichang, 443003 Hubei China; 2 Yichuan Community Health Service Center, Shanghai, 200065 China; 3 Affiliated Renhe Hospital of China Three Gorges University, Yichang, 443001 Hubei China; 4 Institute of Rheumatology, the First College of Clinical Medical Sciences, China Three Gorges University, Yichang, 443003 Hubei China; 5 Third-Grade Pharmacological Laboratory On Chinese Medicine Approved By State Administration of Traditional Chinese Medicine, Medical College of China Three Gorges University, Yichang, 443002 Hubei China

**Keywords:** Sinomenine, Traditional Chinese medicine, Rheumatoid arthritis, Benefit-risk assessment, Multicriteria decision analysis

## Abstract

**Objective:**

A multicriteria decision analysis (MCDA) model was used to evaluate the benefits and risks of traditional Chinese medicine preparations of sinomenine alone or in combination with conventional drugs in the treatment of rheumatoid arthritis (RA) and to provide a basis for the rational clinical application of sinomenine.

**Methods:**

A study search was performed using six major databases, and Review Manager 5.3 was used for data analysis. Then, an MCDA model evaluation system was established for the treatment of RA with sinomenine preparations, and the benefit values, risk values, and total benefit-risk values of sinomenine preparations alone or in combination with conventional drugs were calculated using Hiview 3.2 software. Finally, Monte Carlo simulations were performed using Crystal Ball embedded in Excel software to calculate the 95% confidence intervals (95% CI), and the probability of the differences between the 2 drug regimens was determined to optimize the evaluation results.

**Results:**

Forty-four randomized controlled trials (RCTs) were included. Quantitative assessment of the MCDA model showed that the sinomenine preparation alone offered less benefits than when combined with conventional drugs with a benefit difference of 20 (95% CI 3.06, 35.71). However, the risk of the combination was significantly lower with a risk difference of 13(95% CI -10.26, 27.52). The total value of the benefit-risk of sinomenine alone and in combination with conventional drugs was 46 and 53 at 60% and 40% of the benefit-risk ratio of the two dosing regimens, respectively, with a difference of 7 (95% CI -4.26, 22.12). The probability that the comprehensive score of the combined regimen is greater than that of sinomenine alone is 90.1%, and the evaluation was steady.

**Conclusion:**

The benefit-risk of the combined application regimen of sinomenine is greater than that of sinomenine alone.

## Introduction

Rheumatoid arthritis (RA) is a chronic inflammatory autoimmune disease that affects approximately 0.5 ~ 1% of the population and is characterized by painful, swollen joints that can severely impair physical function and quality of life [1]. Current drug therapy options are limited by severe side effects, high costs, inadequate disease delay for most patients, and the potential for treatment effects to diminish over time [2]. In addition, herbal preparations that can simultaneously reduce the toxic effects of methotrexate and its combination with other drugs, such as leflunomide, have become important options in the clinical treatment of RA [3].

Sinomenine is the main active constituent of the plant *Caulis Sinomenii*, which has anti-inflammatory, analgesic, and immunosuppressive effects and can inhibit the development of RA through multiple pathways and targets [4, 5]. *Caulis Sinomenii* was approved by the State Food and Drug Administration of China for treating RA almost 20 years ago [6]. The Chinese patent medicine Zhengqing Fengtongning, which is produced from *Caulis Sinomenii*, has achieved efficacy in clinical practice. A randomized control trial of 80 patients showed that the efficacy of sinomenine in the treatment of RA was 82.6% compared to 70.8% for conventional drugs and that sinomenine was effective in improving joint symptoms and laboratory parameters in RA patients [7]. On the other hand, sinomenine has some toxicity, and a large retrospective study reported an adverse reaction rate of 4.75%, mainly involving the skin and its accessories (41.23%) and the gastrointestinal system (35.53%) [8]. As the benefits and risks of sinomenine in the treatment of RA coexist, there is no uniform standard for evaluating its clinical potential. The efficacy and adverse effects of sinomenine have been evaluated in several papers at home and abroad, but most of the studies were performed using a systematic evaluation. For example, Zeng Cheng [9] and Liu Weiwei [10] analysed a large number of RCTs but failed to combine the benefits and risks and did not comprehensively evaluate the benefits of sinomenine on RA. The final evaluation of its benefits and risks is one-sided.

Therefore, this study used a multicriteria decision analysis (MCDA) to standardize the scoring of various outcome indicators and evaluated the benefits and risks of sinomenine individually or in combination therapy for RA based on real-world RCT data. The benefits and risks of sinomenine in the treatment of RA are taken into account, the scores of each index can be evaluated more intuitively, and the advantages and disadvantages of the drug regimen can be judged from the comprehensive evaluation to help clinicians make a more favourable choice for patients.

## Materials and methods

### Literature search

We searched randomized controlled trials (RCTs) of Chinese medicine preparations of *Caulis Sinomenii* for RA using the CNKI (China National Knowledge Infrastructure), VIP (China Science and Technology Journal Database), WanFang, Embase, PubMed, and Web of Science databases. Keywords, including rheumatoid arthritis, RA, Zhengqing Fengtongning, Sinomenine, RCT, and randomized literature retrieval time, were employed to search for studies published from database establishment to December 2021. The search strategy is shown in Table 1.

**Table 1:** Search strategy

Data base	Search strategy
PubMed	(Zhengqing Fengtongning OR Sinomenine[Title/Abstract]) AND (RA[Title/Abstract] OR rheumatoid arthritis) arthritis hritisarthritis[Title/Abstract])
EMBASE	#1 (Zhengqing Fengtongning or Sinomenine).mp. [mp = title, abstract, full text, caption text]
	#2 (RA or Rheumatoid Arthritis).mp. [mp = title, abstract, full text, caption text]
	#1 AND #2
Web of Science	TS = ((Zhengqing Fengtongning OR Sinomenine) AND (RA OR rheumatoid arthritis))
CNKI	(Zhengqing Fengtongning OR Sinomenine) AND (RA OR rheumatoid arthritis)
Wan Fang	(Zhengqing Fengtongning OR Sinomenine) AND (RA OR rheumatoid arthritis)
VIP	(Zhengqing Fengtongning OR Sinomenine) AND (RA OR rheumatoid arthritis)

### Inclusion criteria

#### Research target

Patients with a definite diagnosis of RA based on diagnostic criteria referring to the 1987 American College of Rheumatology criteria regardless of gender, age, race, or disease activity.

#### Research type

Randomized controlled trial.

#### Interventions

The experimental group applied Chinese patent medicines containing sinomenine alone or in combination. The control group used conventional medicines, including nonsteroidal anti-inflammatory drugs (NSAIDs) and disease-modifying antirheumatic drugs (DMARDs).

#### Outcomes

Total effective rate, including morning stiffness, tender joint count (TJC), swollen joint count (SJC), rheumatoid factor (RF), C-reactive protein (CRP), erythrocyte sedimentation rate (ESR); the adverse effects rate based on gastrointestinal reaction, leukocyte decline, liver function impairment, and skin lesions.

### Exclusion criteria

Animal studies, review literature, literature for which full text was not available, and literature lacking outcome indicators or records of adverse reactions were excluded.

### MCDA model

**Fig. 1:**
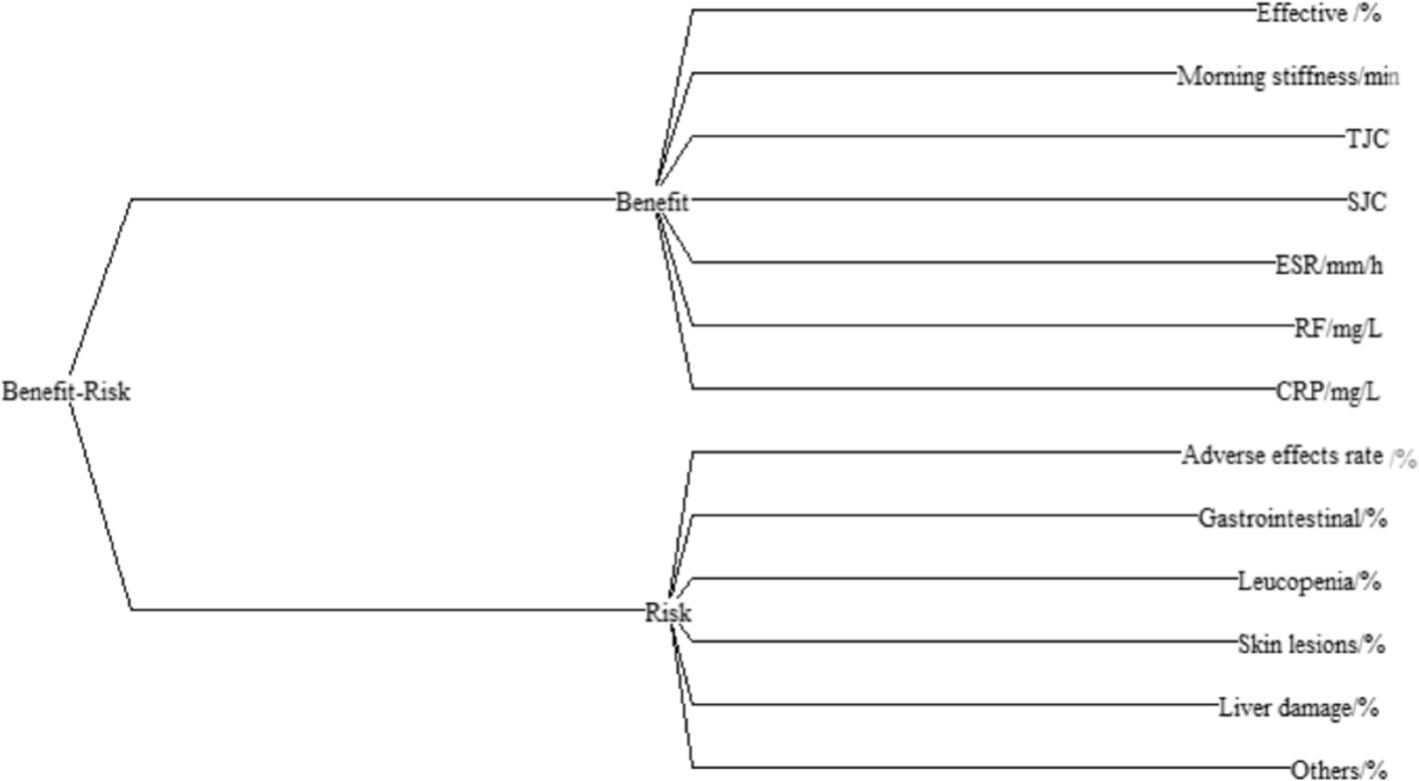
Benefit-risk evaluation effects tree of sinomenine in the treatment of RA. Benefit: Positive outcomes for patients Risk: Negative outcomes for patients TJC: tender joint count SJC: swollen joint count RF: rheumatoid factor CRP: C-reactive protein ESR: erythrocyte sedimentation rate

To establish the decision tree of evaluation indices, the evaluation of outcome indices is divided into benefit and risk indices, and the expression of each index is presented in the form of an effects tree based on Hiview 3.2, as shown in Fig. 1. The SWING method was used to weight each index, and the benefit and risk indices were the primary indices. Due to the special characteristics of RA, we set the benefit weight as 60% and the risk weight as 40%. Here, the symptoms of RA patients mainly consisted of joint pressure pain and joint swelling, so their weight was set as 100. The risk indicators liver function impairment and leukocyte decline were the most serious, so their weights were also set to 100. The weights of the remaining indicators were derived from their respective weights based on comparison with the above indicators, as shown in Table 2.

**Table 2:** Weight and weight score of each indicator for alone and combined

Classification	Outcome indicators	Weight	Best	Worst	Relative weights	Weight Sore	
						**Alone**	**Combine**
Benefit	Effective/%	80	5	1	9.60%	3.456	6.528
	Morning stiffness/min	80	‒13	0	9.60%	2.784	7.776
	TJC	100	‒2	0	12%	5.76	4.92
	SJC	100	‒3	0	12%	6.36	5.04
	ESR/mm/h	40	‒10	0	4.80%	2.496	3.36
	RF/IU/ml	60	‒17	0	7.20%	1.008	4.32
	CRP/mg/L	40	‒7	0	4.80%	1.824	3.504
Benefit Total					60%	23.4	35.4
Risk	Adverse effects Rate /%	80	0	1	8.40%	5.124	3.192
	Gastrointestinal reaction /%	40	0	1	4.20%	2.268	1.638
	Liver damage /%	100	0	1	10.50%	8.085	7.035
	Leucopenia /%	100	0	1	10.50%	5.145	5.775
	Skin lesions /%	40	0	1	4.20%	0	0
	Others/%	20	0	1	2.10%	1.827	1.113
Risk Total					40%	22.4	17.2
Overall Total					100%	46	53

### Data analysis

The benefit and risk indicators of each study were merged using RevMan 5.3 software and are presented in the form of 95% confidence intervals (CIs), as shown in Table 2.

### Scores

Given that the benefit and risk indicators are not measured similarly, comparing their performance in an MCDA requires transforming the data into preference scores. Therefore, based on the single-attribute utility function relationship, we set a 0–100 rating for each of these 13 indicators, For the benefit, a higher preference value indicates a higher gift, whereas the opposite is true for the risk. Some metrics are relatively more important than others, and we give them more weight by weighing them. The optimal value and the worst value for each indicator are specified in Table 2.

### Sensitivity

Due to the subjective nature of the weight assigned to each indicator, a sensitivity analysis is required to demonstrate the stability of its weight selection. If the weight changes by greater than 20%, the evaluation results will change. Then, the weighting of indicators is more reasonable, and the sensitivity established by the MCDA model is not high and more stable, and vice versa [11].

### Monte Carlo simulation

As the benefit and risk values obtained by RevMan 5.3 are point estimates, Monte Carlo simulations were performed in Excel via Crystal Ball 11 to iteratively calculate the benefit and risk values and the 95% CI of the total benefit-risk values for the 2 groups (sinomenine preparation alone and in combination) and to simulate the probability of differences between the 2 groups to optimize the MCDA evaluation results.

## Results

### Include studies

**Fig. 2:**
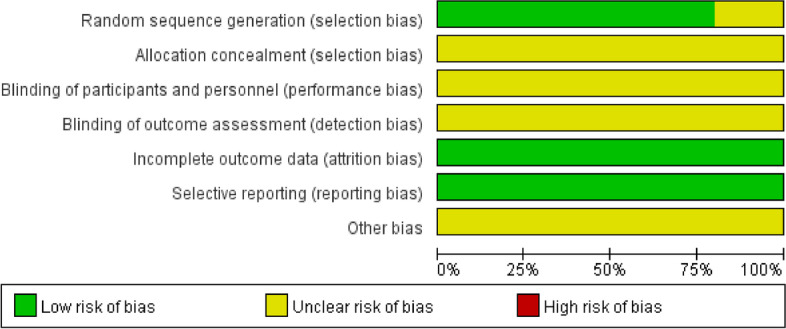
Publication bias of included literature

A total of 1163 articles were retrieved for the first time. After removing duplicates and noncompliant studies, 44 studies were finally obtained [12–55], all of which were Chinese RCTs. The studies were published from 1999 to 2021. Among them, 14 articles reported sinomenine use alone. Thirty-one articles included combined treatments, and all but 9 studies mentioned the random assignment method. Bias in data integrity and selective reporting was not observed in any of the studies, and the blinding method or distribution method of the participants was not reported in any of the studies, as shown in Fig. 2.

### Consolidation result

A meta-analysis of 14 studies showed that the sinomenine preparation alone was effective in improving joint symptoms (morning stiffness, joint tenderness, joint swelling and pain) and laboratory parameters (ESR, RF, CRP) and had a lower incidence of adverse reactions than conventional drugs. However, a high incidence of skin damage was reported. The meta-analysis of 30 combined drugs showed that sinomenine alone could effectively improve patients' joint symptoms, laboratory indices, and the incidence of adverse reactions compared with the ordinary medicine group, but the incidence of allergic reactions was still greater than that in the conventional drug group (Table 3).

**Table 3:** Merger results of alone and combined of each indicator

	**Indicator**	**Alone**		**Combined**	
		**RCT**	**Result**	**RCT**	**Result**
Benefit	Effective /%	11	2.44;95%*CI*(1.87,3.19),*P*< 0.00001	20	3.7;95%CI(2.72,5.02),*P*< 0.00001
	Morning stiffness /min	13	‒3.83;95% *CI* (‒4.87, -2.8),*P*< 0.00001	20	‒10.51;95%*CI* (‒12.72,-8.31),* P*< 0.00001
	TJC	13	‒0.95;95% *CI* (‒1.39, -0.51),* P*< 0.00001	17	‒0.82;95%*CI* (‒1.6,-0.03),* P* = 0.04
	SJC	13	‒1.65;95% *CI* (‒2.1, -1.19),* P*< 0.00001	17	‒1.25;95% *CI* (‒1.98, -0.52),* P* = 0.0008
	ESR/mm/h	11	‒5.15;95% *CI* (‒7.98, -2.23),* P*< 0.0004	26	‒6.96;95% *CI* (‒9.65, -4.27),* P* < 0.00001
	RF/IU/ml	9	‒2.36;95% *CI* (‒3.25, -1.48),* P* < 0.00001	22	‒10.18;95% *CI* (‒16.4, -3.97),* P* = 0.001
	CRP/mg/L	8	‒2.63;95% *CI* (‒5.49, -0.24),* P* = 0.07	19	‒5.14;95% *CI* (‒6.63, -3.66),* P*< 0.00001
Risk	Adverse effects Rate /%	6	0.39;95%*CI* (0.21,0.72),* P* = 0.003	20	0.62;95%*CI* (0.43,0.89),* P* = 0.008
	Gastrointestinal reaction /%	12	0.46;95%*CI* (0.19,1.12),* P* = 0.09	19	0.61;95%*CI* (0.43,0.85),* P* = 0.003
	Liver damage /%	6	0.23;95%*CI* (0.07,0.73),* P* = 0.03	15	0.45;95%*CI* (0.26,0.78),* P* = 0.002
	Leucopenia /%	8	0.51;95%*CI* (0.18,1.44),* P* = 0.2	17	0.48;95%*CI* (0.29,0.78),* P* = 0.003
	Skin lesions /%	9	1.54;95%*CI* (0.61,3.89),* P* = 0.36	20	1.4;95%*CI* (0.88,2.23),* P* = 0.16
	Others/%	7	0.13;95%*CI* (0.04,0.38),* P* = 0.0002	8	0.47;95%*CI* (0.25,0.88),* P* = 0.02

### Comprehensive benefits

**Fig. 3:**
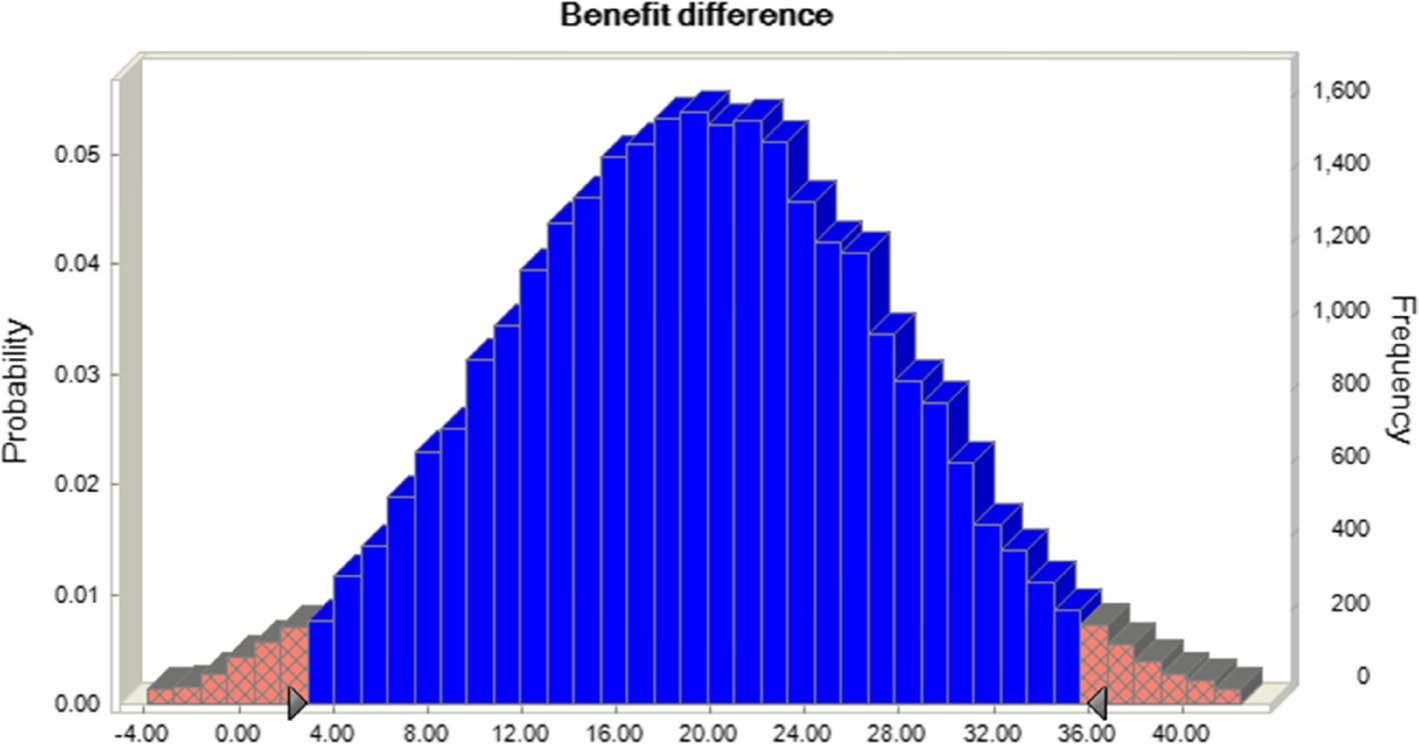
Differential benefits between alone and combined use

The benefit values of a sinomenine preparation alone and combined with ordinary medicine were 39 and 59, respectively. Thus, sinomenine combination therapy has a better effect on RA. However, sinomenine use alone is more effective in alleviating joint tenderness and joint swelling, as shown in Table 2. Monte Carlo simulation was performed using crystal ball software and 30,000 iterations. The results showed that the difference between sinomenine alone and combined with conventional medicines was 20 (95% CI 3.06, 35.71), as shown in Fig. 3.

### Comprehensive risk

**Fig. 4:**
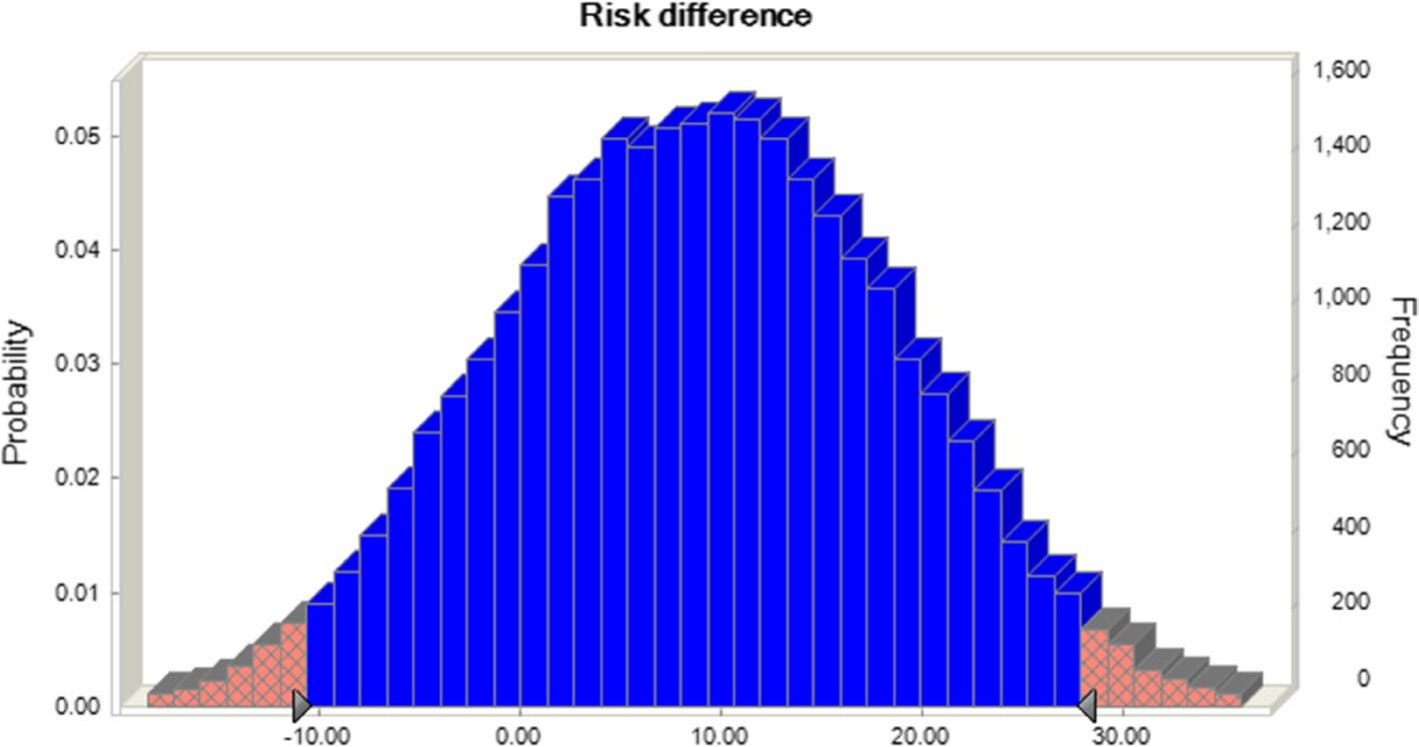
The deviation of risk between alone and combined

The risk values of sinomenine alone and in combination were 56 and 43, respectively, demonstrating that the risk of sinomenine alone for RA is lower, especially in terms of damage to liver function. The incidence of adverse reactions to sinomenine alone is more advantageous, but both regimens scored 0 in skin damage reactions, indicating that sinomenine is not skin friendly (see Table 2). Monte Carlo simulation iterations were performed, and the results showed that the difference between sinomenine alone and in combination with conventional medicines was 13 (95% CI -10.26, 27.52), as shown in Fig. 4.

### Comprehensive benefits-risk

**Fig. 5:**
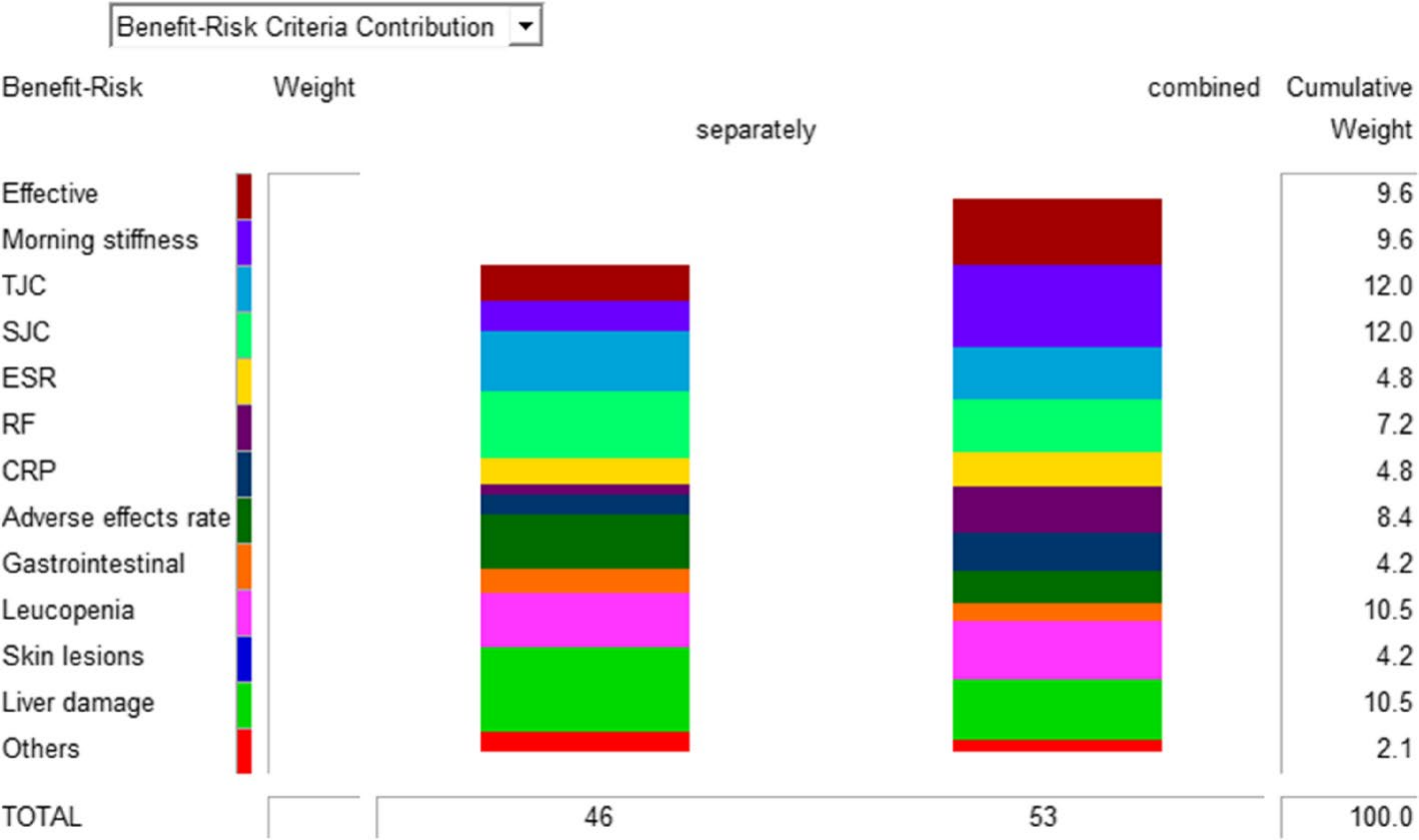
Benefits—risks of alone and combined

**Fig. 6:**
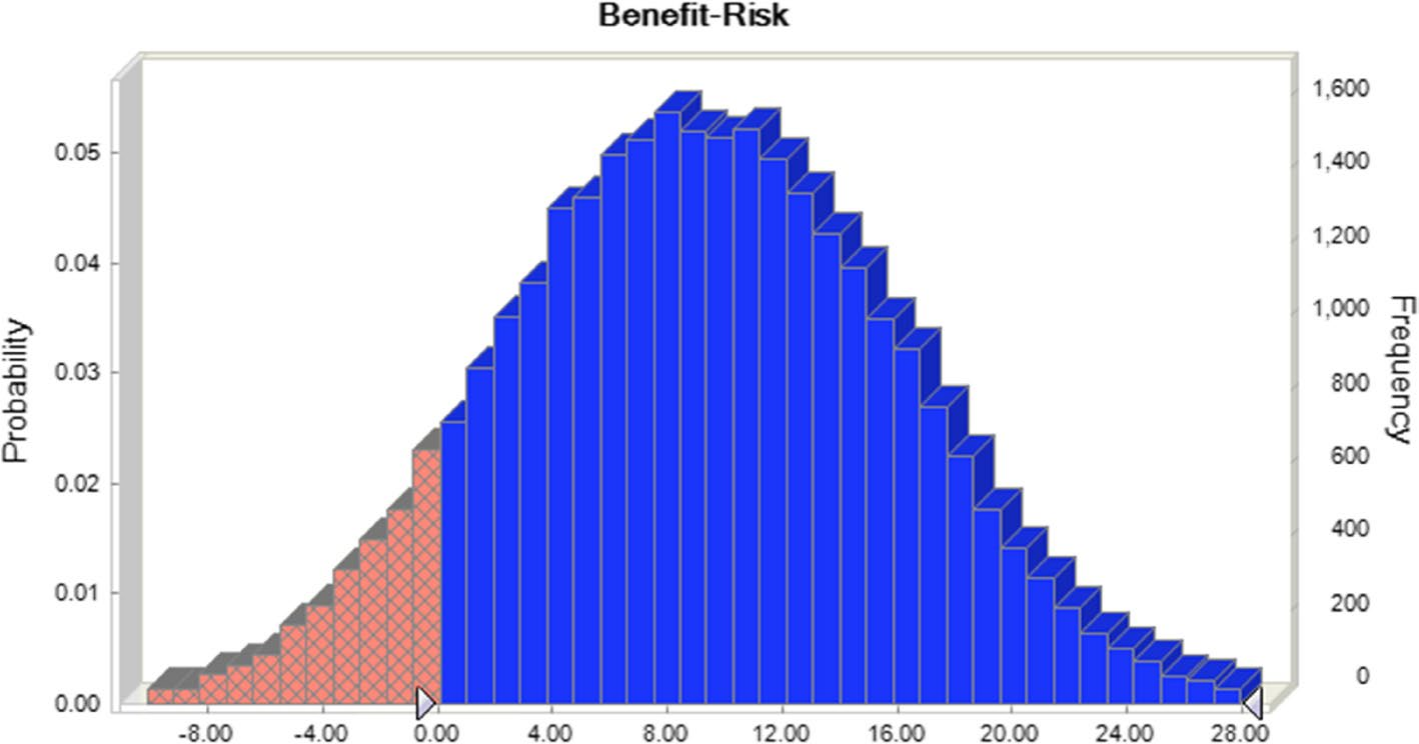
The probability of combined use being better than alone

The benefit-risk values for sinomenine alone and the combination were 46 and 53, respectively, and the benefit-risk for the combination was sevenfold greater than that for sinomenine alone. The combined use scored is relatively evenly distributed in all areas, indicating that the regimen can alleviate the patient's condition via numerous mechanisms. Thus, the patient can benefit more. Regarding hepatic impairment and the incidence of adverse reactions, the score was higher for sinomenine alone. Considering that the patients had liver disease, perhaps sinomenine alone was more appropriate (Fig. 5). Monte Carlo simulation showed that the total benefit-risk value difference between the 2 drug regimens was 7 (95% CI -4.26, 22.12), as shown in Fig. 6. After 30,000 simulations, the probability that the combined regimen would score higher than the single agent regimen was 90.1% (Fig. 6).

### Sensitivity analysis

**Fig. 7:**
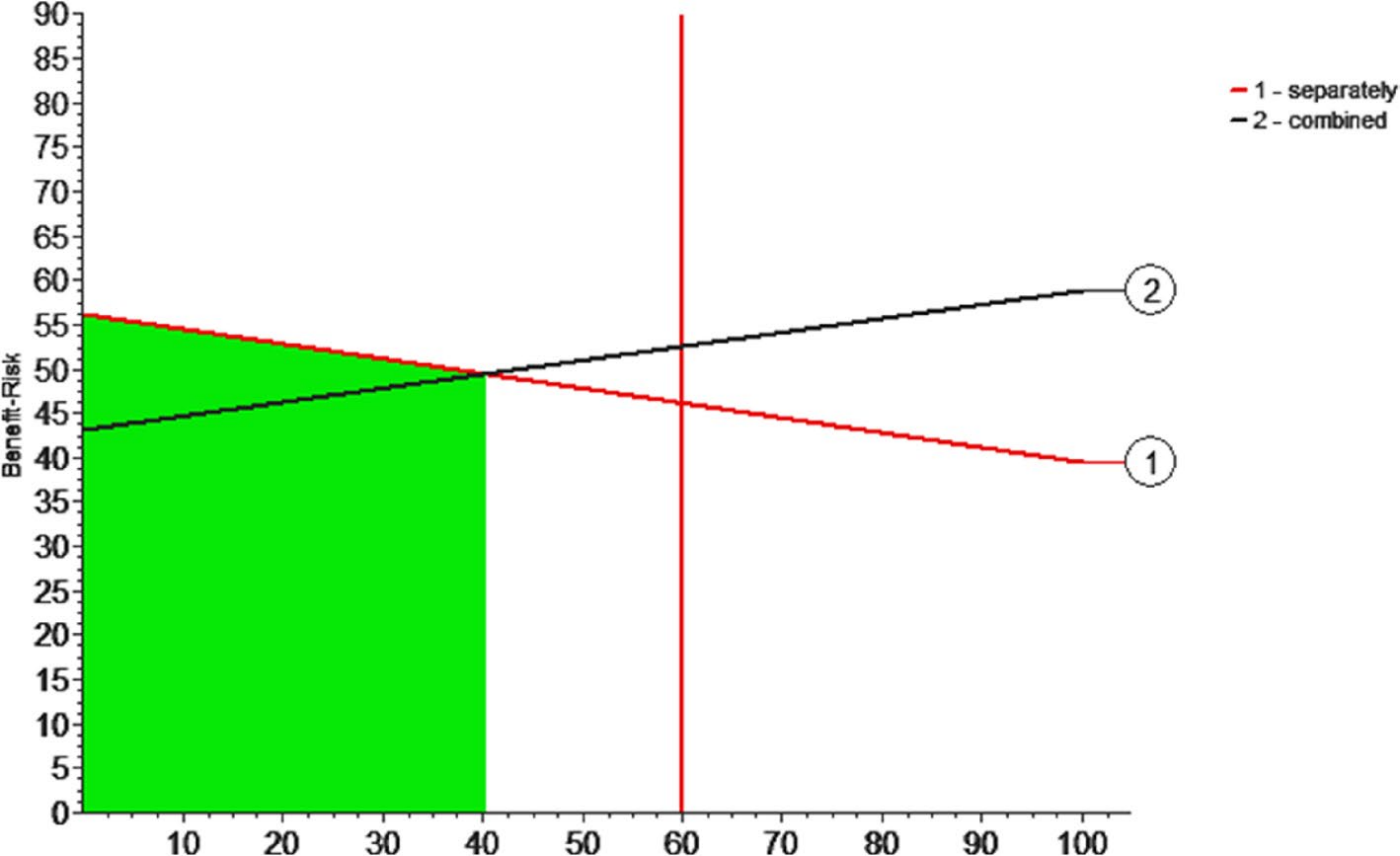
Sensitivity analysis of benefit-risk

When the relative benefit weight is 60%, the total benefit-risk for the combination of sinomenine is greater than that for sinomenine, as shown in Fig. 7. The above evaluation results change when the comparative benefit weight is reduced to less than 40%. As mentioned above, the evaluation results change when the change range of the weight exceeds 20%, indicating that the current subjective weight setting is reasonable. Thus, the weight assigned to the benefit-risk in this study is affordable, and the model has high stability.

## Discussion

The MCDA model provides a structured framework within which the aspects considered to be important to the overall aspects are assessed and graded, and specific judgements are made. The model is mainly comprised of three steps: determine the main influencing factors, evaluate the evidence for each factor, and ensure that the results of the MCDA model are meaningful. In this study, the hypothesis was artificially graded twice, and the relative weight of relevant outcome indicators was obtained. Although the constituent ratios and established weights are subjective, the assumptions underlying their use are clear. Theoretical and clinical perspectives work together to determine what represents value and facilitate drug decisions. MCDA is also widely used in the benefit-risk assessment of drugs. For example, MCDA is used to identify the most suitable treatment for patients with NVAF [56] and to perform a benefit-risk assessment of six over the-counter analgesics [57]. In addition, we performed Monte Carlo simulations based on MCDA. Randomized simulations were used to compare the benefits and risks of the two treatment options. The accuracy of the results was verified theoretically, and the benefits and risks of the two treatments were further verified.

### Efficacy

RA is a chronic inflammatory autoimmune disease influenced by both genetic and environmental factors [58]. As there is no cure for RA, the treatment goals are to reduce pain and stop/slow further damage [59]. *Caulis linoleum* has been utilized to treat RA for more than 1,000 years as recorded in a compendium of material media. Many recent studies have shown that it has a significant effect on RA [60, 61], and can effectively improve the amount of joint swelling, morning stiffness time, ESR, RF and other patient signs and laboratory examination indicators.

These features are observed in the RCTs included in this study. Whether used alone or in combination, it has better efficacy than conventional drugs in improving patient signs and laboratory indicators, especially in combination. Monte Carlo simulation showed that sinomenine combined with conventional drugs was 100% better than sinomenine alone and more effective in reducing morning stiffness time and RF. This finding indicates that the combination of sinomenine with conventional drugs may achieve better clinical efficacy and is more favourable for alleviating patients' conditions, providing a reliable theoretical basis for clinical medicine.

### Risk

Due to the chronic increase of RA patients and the need for long-term medication, the risk of drugs is at the center of the attention of clinicians and patients. Traditional DMARD conventional drugs work slowly, and many months are needed to reach the effect of the control condition; however, the cumulative effect of long-term medication can lead to various complications, such as gastrointestinal reactions, liver and kidney function, and bone marrow toxicity. Studies have demonstrated that small doses of MTX could also lead to gastrointestinal tract, infection, lung diseases, and other adverse reactions [62]. Similarly, sinomenine also has unfavourable reactions. Adverse reactions caused by sinomenine mainly include rash and gastrointestinal discomfort, which may be related to strong histamine release [63], therefore, risk assessment is also essential.

Our study showed a series of unfavourable reactions to sinomenine. Skin damage was reported most frequently. The merged data showed that both were below the minimum criteria we set, so both regimens scored 0. However, from the perspective of the included RCT, its adverse reactions and system damage were less than those of conventional Western medicine drugs either alone or in combination. Among them, the incidence of adverse reactions of a single application was lower, and the scores of multiple adverse reactions were better than those of a combined regimen. Therefore, patients with poor tolerance in clinical application may choose to use sinomenine alone to obtain more benefits.

### Benefit-risk

Sinomenine has both benefits and risks in the treatment of RA. Therefore, comprehensive consideration should be paid to the application of sinomenine in the treatment of RA, and multiple factors should jointly determine the medication regimen to improve drug compliance. Although numerous studies have analysed the effectiveness and safety of sinomenine, these studies have neglected to unify the benefit and risk evaluation and failed to comprehensively evaluate its overall utility for RA patients, which is one-sided to some extent. Based on the MCDA model and Monte Carlo simulation, this study further verified the theoretical differences in benefits and risks between the two treatment regimens. In the analysis of this study, the weights of benefit and risk were set at 60% and 40%, respectively. The reason for this allocation is that compared to side effects, limb deformities, joint swelling and pain have more far-reaching and serious consequences for patients. Moreover, sensitivity analysis showed that our allocation was reasonable. When performing the sensitivity analysis, we observed a significant change in the results when the benefit weight was decreased to 40%, indicating that the weight assigned was reasonable according to the previous statement. The final results demonstrate that the two treatment regimens differ in their benefit and risk scores. Specifically, sinomenine in combination with conventional drugs offered more benefits in the treatment of RA compared with sinomenine alone. However, the combination regimen exhibited higher risk scores than sinomenine alone, which may be related to the inherent risk of combining drugs. Combining the benefits and risks, the combination of sinomenine with conventional drugs has a higher score, and the combination regimen is more recommended than sinomenine alone for clinical applications.

### Bias

A total of 44 literatures were included in this study. According to the risk assessment results, there is a certain deviation risk, and the main risk factors are blind design and selection allocation hiding. It is difficult to avoid the bias caused by the lack of rigor in the research program. Only high-quality research program design can alleviate this situation, but there is still room for improvement in the blind design of such studies. Secondly, sinomenine is only applied in the Chinese market at present, so the studies we included were all in Chinese, which may also lead to the deviation of the results. The studies we included ranged from 1999 to 2021, and there may be some bias between individual studies. To sum up, we still need to design rigorous research schemes and higher quality studies to further verify the correctness of our research.

## Conclusion

In summary, the combination regimen may be more beneficial to patients than sinomenine alone. The incidence of adverse reactions for sinomenine alone is lower than that noted for the combination regimen, and sinomenine alone may be more appropriate for patients with poor health status or allergies. This study is based on the MCDA model that provides a clear method of assigning weights, and the above conclusions were obtained after several iterations of simulations. This information is expected to serve as a reference for clinical practice.

## Data Availability

The data used to support the findings of this study are available from the corresponding author upon request.

## Abbreviations

RA: Rheumatoid arthritis
RCT: Randomized control trial
MCDA: Multi-criteria decision analysis
CNKI: China national knowledge infrastructure;
VIP: China science and technology journal database
NSAIDs: Non-steroidal anti-inflammatory drugs
DMARDs: Disease-modifying antirheumatic drugs
TJC: Tender joint count
SJC: Swollen joint count
RF: Rheumatoid factor
CRP: C-reactive protein
ESR: Erythrocyte sedimentation rate
CIs: Confidence intervals
MTX: Methotrexate

## References

[1] Sparks JA (2019). Rheumatoid Arthritis. Ann Intern Med.

[2] de Seabra Rodrigues Dias IR, Lo HH, Zhang K, Law BYK, Nasim AA, Chung SK, Wong VKW, Liu L (2021). Potential therapeutic compounds from traditional Chinese medicine targeting endoplasmic reticulum stress to alleviate rheumatoid arthritis. Pharmacol Res.

[3] Sun Y, Huang Y, Chen T, Li X, Chen J, Wang Z, Lin K, Gao Y, He L (2020). Effect of downregulation of serum MMP-3 levels by traditional Chinese medicine ingredients combined with methotrexate on the progression of bone injury in patients with rheumatoid arthritis: A protocol for a systematic review and meta-analysis. Medicine.

[4] Guo X, Ji J, Feng Z, Hou X, Luo Y, Mei Z (2020). A network pharmacology approach to explore the potential targets underlying the effect of sinomenine on rheumatoid arthritis. Int Immunopharmacol.

[5] Shen Q, Zhang X, Qi J, Shu G, Du Y, Ying X (2020). Sinomenine hydrochloride loaded thermosensitive liposomes combined with microwave hyperthermia for the treatment of rheumatoid arthritis. Int J Pharm.

[6] Xu W, Chen S, Wang X, Wu H, Tahara K, Tanaka S, Sugiyama K, Yamada H, Sawada T, Hirano T (2021). Effects of sinomenine on the proliferation, cytokine production, and regulatory T-cell frequency in peripheral blood mononuclear cells of rheumatoid arthritis patients. Drug Dev Res.

[7] Cheng Y (2013). Clinical effect of Zhengqing Fengtongning tablet on 80 cases of rheumatoid arthritis. China Health Industry.

[8] Wu Z, Long L, Chen X, Chen G (2016). Analysis of 193 Cases of Adverse Drug Reactions Induced by Zhengqingfengtongning Sustained Release Tablets. Chin J Pharmacovigilance.

[9] Zeng C, Shuai YF, Li X (2021). Meta-analysis of efficacy and safety of sinomenine combined with methotrexate in treatment of rheumatoid arthritis. Zhongguo Zhong Yao Za Zhi.

[10] Liu W, Qian X, Ji W, Lu Y, Wei G, Wang Y (2016). Effects and safety of Sinomenine in treatment of rheumatoid arthritis contrast to methotrexate: a systematic review and Meta-analysis. J Tradit Chin Med.

[11] Mussen F, Salek S, Walker S (2007). A quantitative approach to benefit-risk assessment of medicines - part 1: the development of a new model using multi-criteria decision analysis. Pharmacoepidemiol Drug Saf.

[12] Zhu J, Chen P (2001). 30 cases of rheumatoid arthritis treated by integrated Chinese and Western medicine. Fujian J Tradit Chin Med.

[13] Ji H, Zhu Z (2006). 30 cases of rheumatoid arthritis treated with low dose Zhengqing Fengtongning combined with methotrexate. Herald of Med.

[14] Yang B, Liu X (2015). The Clinical Curative effect of Zhengqingfengtongning Combined with Methotrexate in Treatment of Rheumatoid Arthritis. China J Pharma Econ.

[15] Zhang J, He Y, Shi S (2019). Clinical effect of Zhengqing Fengtongning combined with Yunke, methotrexate and hydroxychloroquine on rheumatoid arthritis and their influence on ESR, RF, CRP and CCP. Chinese J Disaster Med.

[16] Chen Z, Wang X (2021). Clinical Efficacy of Zhengqing Fengtongning Combined with Methotrexate in the Treatment of Rheumatoid Arthritis. Chin Foreign Med Res.

[17] Wu Y (2003). Clinical observation of 40 cases of rheumatoid arthritis treated with low dose methotrexate and Zhengqing Fengtongning. J New Chinese Med.

[18] Gu J, Wang W, Ma Y (2013). Clinical observation of 58 cases of active rheumatoid arthritis treated by Zhengqing Fengtongning. Guiding J Tradit Chin Med Pharmacy.

[19] Xia Y, Tu S, Hu Y, Wang Y, Chen Z, Liu Y (2012). Clinical Observation of Effect of Methotrexate Combined with Zhengqingfengtongning in Treating Active Rheumatoid Arthritis Based on Syndrome Differentiation. Res Integ Trad Chin West Med.

[20] Wang W (2010). Clinical observation of methotrexate combined with Zhengqingfengtongning in treating 120 rheumatoid arthritis. Zhejiang Pract Med.

[21] Lu Y, Su J (2011). Clinical observation of Zhengqing Fengtongning combined with methotrexate in the treatment of rheumatoid arthritis. Liaoning J Tradit Chin Med.

[22] Yu Y, Chi S (2005). Clinical observation of Zhengqing Fengtongning combined with sulfasalazine in the treatment of rheumatoid arthritis. Clin Med China.

[23] Hu X, Wu D, Tan G, Hu G, Sun K (1999). Clinical observation of zhengqing Fengtongning in the treatment of 428 cases of rheumatoid arthritis. Hunan J Tradit Chin Med.

[24] Zhang F, Li J (2011). Clinical observation of zhengqing Fengtongning sustained-release Tablet combined with Western medicine in treating 53 cases of active rheumatoid arthritis. Guiding J Tradit Chin Med Pharm.

[25] Sun R, Lin Z (1999). Clinical observation on 38 cases of rheumatoid arthritis treated by "Zhengqing Fengtongning". Shanghai J Tradit Chin Med.

[26] Zhang Q (1998). Clinical observation on 52 cases of rheumatoid arthritis treated by Zhengqing Fengtongning. Fujian J Tradit Ch Med.

[27] Yang X, Yang J (2003). Clinical observation on 118 cases of rheumatoid arthritis treated by Zhengqing Fengtongning. J Chinese Phys.

[28] Huang Z, Wang N (2010). Clinical observation on treatment of rheumatoid arthritis with zhengqing fengtongning retard tablets. Hebei Med J.

[29] Liu W, Liu X, Liu B (2006). Clinical observation on treatment of rheumatoid arthritis with Zhengqing Fengtongning Retard Tablets :a report of 60 cases. J Chin Integ Med.

[30] Zhang Z, Shen J, Liu K. Clinical observation on treatment of senile rheumatoid arthritis by Zhengqing Fengtongning. J Tradit Chin Orthop Traumatol. 2002;14(02):12–13+63.

[31] Ma H, Liang X (2020). Clinical Observation on Zhengqing Fengtongning Sustained-Release Tablets Combined with Methotrexate in the Treatment of Rheumatoid Arthritis. Chin Med Modern Distance Educ Cina.

[32] Xu C (2006). Clinical Observation on Zhengqing-Fengtongn inTablets for theTreatment of 80 Cases of Rheumatoid Arthritis. Guiding J Tradit Chin Med Pharm.

[33] Lin X, Cai X, Ye J (2009). The clinical observition on Sinomenine for Rheumatoid Arthritis. J Hunan Unive Chinese Med.

[34] Shen X, Li H (2005). Clinical studies on Zhengqingfengtong ning in treatment of rheumatoid arthritis. J Anhui Tradit Chin Med College.

[35] Ling Y, Wang N, Feng H, Xie J, He D (2014). Clinical study of sinomenine combined with methotrexate on the repair of bone destruction in rheumatoid arthritis. World Clin Drugs.

[36] Gu F, Sun Y, Chen S, Wang P, Ding C, Sun L (2014). Clinical study of sinomenine in combination with methotrexate for the treatment of active rheumatoid arthritis. Shanghai J Tradit Chin Med.

[37] Huang G, Li J, Huang Y, Huang D, Tang L, Zhang S, Li Q. The Clinical Study on Sinomenine for100 Patients with Rheumatoid Arthritis. J Emerg Tradit Chin Med. 2007;16(04):416–417+421.

[38] Zhang L (2020). Clinical Study on the Effect of Zhengqing Fengtongning on the Disease Activity of Patients with Rheumatoid Arthritis. Chin Foreign Med Treatment.

[39] Lao Z (2000). Combination of sinomenine and methotrexate in the treatment of rheumatoid arthritis. Chin J New Drugs Clin Remed.

[40] Li C, Gao Y, Fan C, Chen Q (2005). Comparative observation of clinical efficacy of Zhengqing Fengtongning and Tripterygium wilfordii glycosides in treating rheumatoid arthritis. The Med Forum.

[41] Gao J, Zhang Q, Ji N, Deng D, Wang H, Tong D (1999). Comparison of sinomenine alone or with chloroquine and Tripterygium glycosides in the treatment of rheumatoid arthritis. Chin J New Drugs and Clin Remed.

[42] Li D (2018). Effect of leflunomide combined with sinomenine in the treatment of rheumatoid arthritis. J Clin Med.

[43] Liu Y, Huang Y, Lin S, Pan C, Zhang Y, Yang Z, Zhan Y (2020). Effect of Zhengqingfengtongning Tablet Combined with Celecoxibon Disease Activity Indexes, Inflammatory Factors and Red Blood Cell Immune Function in Patients with Rheumatoid Arthritis. Progress in Modern Biomed.

[44] Li Y, Feng X, Zhang S, Li C (2008). Effects of sinomenine combined with methotrexate on rheumatoid arthritis. Pract Pharm Clin Remed.

[45] Dai L, Song X, Wang J (2018). Effects of sinomenine combined with methotrexate on serum inflammatory factors in patients with rheumatoid arthritis and its safety. Shaanxi Med J.

[46] Ke S, Shi W (2020). The Efficacy Evaluation of Sinomenine Hydrochloride Enteric-coated Tablets Combined with Meloxicam and Methotrexate in the Treatment of Rheumatoid Arthritis. Chin Foreign Med Res.

[47] Cai Q, Jin S, Chen G, Yue T (2019). Efficacy of sinomenine combined with methotrexate on early rheumatoid arthritis and its influence on expressions of MMP-3 and RANKL/OPG. Acad J Shanghai Univ Tradit Chin Med.

[48] Yang D, Zheng X (2005). Observation on the curative effect of Zhengqing Fengtongning sustained-release tablets in the treatment of rheumatoid arthritis. Drug evaluation.

[49] Su J (2013). A Randomized Controlled Ctudy of Cinomenine Custained - release Tablets Combined With Western Medicine in the Treatment of Rheumatoid Arthritis. J Pract Tradit Chin Intern Med.

[50] Zhang G, Fu Z, Wang K, Liu X (2005). The study of Zhengqinfengtongning on expression of chemokine RANTES in patients with rheumatoid arthritis. Proceed Clin Med.

[51] Sun S, Lan H (2006). Summarization on theTreatment of 62 Cases of Rheumatoid Arthritis WithZhengqinfengtongninTablets and Methopterin. Guiding J Tradit Chin Med Pharm.

[52] Hu Q, Guan X, Lian Y, Lei L, Tao L (2010). Therapeutic Effect Observations of Leflunomi de and Methopterin Combined with Zhengqing Fengtongning in Treat ment of Rheumatoid Arthritis. J Modern Clin Med.

[53] Yang C (2009). Therapeutic effect of methotrexate combined with Traditional Chinese medicine in the treatment of rheumatoid arthritis. Chin Prim Health Care.

[54] Wu H, Wang Y (2014). Total Glucosides of Paeony Combined with Total Glucosides of Paeony Combined with Zhengqing Fengtongning Treat RA(Rheumatoid Arthritis) in Active Stage. J Zhejiang Chin Med Univ.

[55] Lin Z, Dai L, Zheng L, Dai Y, Li T (2007). Value of “ZhengqingFengtongning”onTreatment of Rheumatoid Arthriti. Clin J Med Officer.

[56] Mendoza-Sanchez J, Silva F, Rangel L, Jaramillo L, Mendoza L, Garzon J, Quiroga A (2018). Benefit, risk and cost of new oral anticoagulants and warfarin in atrial fibrillation; A multicriteria decision analysis. PLoS One.

[57] Moore A, Crossley A, Ng B, Phillips L, Sancak Ö, Rainsford KD (2017). Use of multicriteria decision analysis for assessing the benefit and risk of over-the-counter analgesics. J Pharm Pharmacol.

[58] Croia C, Bursi R, Sutera D, Petrelli F, Alunno A, Puxeddu I (2019). One year in review 2019: pathogenesis of rheumatoid arthritis. Clin Exp Rheumatol.

[59] Bullock J, Rizvi SAA, Saleh AM, Ahmed SS, Do DP, Ansari RA, Ahmed J (2018). Rheumatoid Arthritis: A Brief Overview of the Treatment. Med Princ Pract.

[60] Huang RY, Pan HD, Wu JQ, Zhou H, Li ZG, Qiu P, Zhou YY, Chen XM, Xie ZX, Xiao Y (2019). Comparison of combination therapy with methotrexate and sinomenine or leflunomide for active rheumatoid arthritis: A randomized controlled clinical trial. Phytomedicine.

[61] Liu W, Zhang Y, Zhu W, Ma C, Ruan J, Long H, Wang Y (2018). Sinomenine Inhibits the Progression of Rheumatoid Arthritis by Regulating the Secretion of Inflammatory Cytokines and Monocyte/Macrophage Subsets. Front Immunol.

[62] Kour G, Haq SA, Bajaj BK, Gupta PN, Ahmed Z (2021). Phytochemical add-on therapy to DMARDs therapy in rheumatoid arthritis: In vitro and in vivo bases, clinical evidence and future trends. Pharmacol Res.

[63] Huang L, Dong Y, Wu J, Wang P, Zhou H, Li T, Liu L (2017). Sinomenine-induced histamine release-like anaphylactoid reactions are blocked by tranilast via inhibiting NF-kappaB signaling. Pharmacol Res.

